# SIRT7: a sentinel of genome stability

**DOI:** 10.1098/rsob.210047

**Published:** 2021-06-16

**Authors:** Ming Tang, Huangqi Tang, Bo Tu, Wei-Guo Zhu

**Affiliations:** ^1^ Clinical and Translational Research Center, Shanghai Key Laboratory of Maternal-Fetal Medicine, Shanghai First Maternity and Infant Hospital, Tongji University School of Medicine, Shanghai 201204, People's Republic of China; ^2^ Guangdong Key Laboratory of Genome Instability and Human Disease Prevention, Shenzhen University International Cancer Center, Marshall Laboratory of Biomedical Engineering, Department of Biochemistry and Molecular Biology, Shenzhen University School of Medicine, Shenzhen 518055, People's Republic of China; ^3^ Fred Hutchinson Cancer Research Center, Seattle, WA 98101, USA

**Keywords:** SIRT7, genome stability, DNA repair, ageing, cancer

## Abstract

SIRT7 is a class III histone deacetylase that belongs to the sirtuin family. The past two decades have seen numerous breakthroughs in terms of understanding SIRT7 biological function. We now know that this enzyme is involved in diverse cellular processes, ranging from gene regulation to genome stability, ageing and tumorigenesis. Genomic instability is one hallmark of cancer and ageing; it occurs as a result of excessive DNA damage. To counteract such instability, cells have evolved a sophisticated regulated DNA damage response mechanism that restores normal gene function. SIRT7 seems to have a critical role in this response, and it is recruited to sites of DNA damage where it recruits downstream repair factors and directs chromatin regulation. In this review, we provide an overview of the role of SIRT7 in DNA repair and maintaining genome stability. We pay particular attention to the implications of SIRT7 function in cancer and ageing.

## Graphical abstract

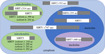

## 1. Introduction

The integrity and stability of the genome are constantly challenged by both intrinsic or extrinsic insults such as replication stress, oxidative damage, ultraviolet light, ionizing radiation and various genotoxic reagents, which can ultimately lead to DNA damage [1]. If DNA damage is not properly repaired, it can result in diseases such as cancer, or pathologies associated with ageing [2,3]. To counteract DNA damage, cells have evolved an elaborate mechanism—a tightly regulated DNA damage response (DDR) that detects, signals and repairs DNA lesions. Both normal and malignant cells depend on various DDR pathways to protect their genomes [4]. Depending on the cell cycle stage, genetic background and types of DNA damage, there are five major repair pathways, including non-homologous end joining (NHEJ), homologous recombination (HR), mismatch repair (MMR), base excision repair (BER) and nucleotide excision repair (NER) [5].

Post-translational modifications have a crucial role in mediating the cellular response to DNA damage, providing a means of changing protein activity without the necessity of *de novo* protein synthesis [6]. The most common post-translational modifications include phosphorylation, ubiquitination, acetylation, methylation and sumoylation [6]. These modifications are reversible due to their regulation by two opposing enzymes. For example, lysine residues are acetylated due to the activity of acetyltransferases that attach acetyl groups and deacetylated due to the activity of histone deacetylases (HDACs) [7].

**Figure 1.**
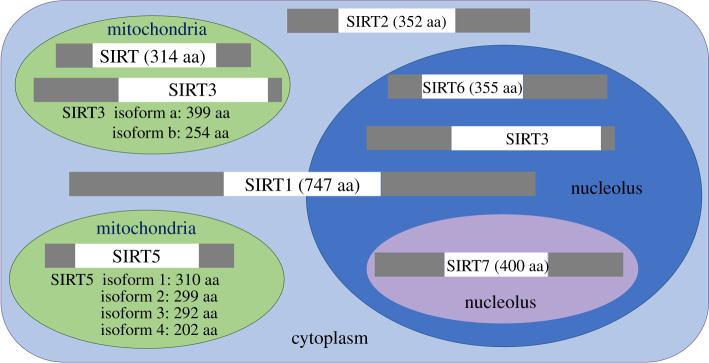
Sirtuin family protein structures and subcellular localizations.

In higher eukaryotes, HDACs can be divided into four classes. Class I Rpd3-like enzymes are comprised of HDAC1, 2, 3 and 8. Class II Hda1-like enzymes are composed of HDAC4, 5, 6, 7, 9 and 10. Class III Sir2-like enzymes consist of seven sirtuins, SIRT1-7, which depend on NAD^+^ as a coenzyme. Class IV contains only HDAC11 [7–9]. Sirtuins are a class of deacetylases that are homologous to Sir2 (silent information regulator 2), seven members of this family, SIRT1-7, all have a conserved catalytic domain (figure 1). In addition to homology, sirtuins have different types of enzyme catalytic activities, such as ADP ribosyl transferase, desuccinylase and demalonylase, the diverse enzyme activities endow sirtuins with diverse biological functions [10–12].

Among the sirtuins, SIRT7 is the least studied protein, but recent breakthroughs have shown that it is also involved in multiple cellular processes and its biological function is gradually becoming clear. In this review, we outline the current studies regarding the role of SIRT7 in DDR and its potential therapeutic role in disease.

## 2. SIRT7 structure and function

SIRT7 encodes a 400 amino acid protein and in humans' functions as an NAD^+^-dependent class III histone deacetylase [13]. Compared with other nuclear-localized sirtuins (SIRT1 and SIRT6), SIRT7 exhibits deacetylase, desuccinylase and deglutarylase activities [14–16]. Over the past two decades, several SIRT7 substrates have been identified (table 1). The wide variety of SIRT7 substrates suggests that SIRT7 participates in diverse biological processes.

**Table 1.** SIRT7 targets.

substrate	activity	functions
p53	deacetylation	apoptosis, heart hypertrophy and inflammatory cardiomyopathy [17]
H3K18	deacetylation	oncogenic transformation [14]
DAF-16	deacetylation	stress response [18]
PAF53	deacetylation	pre-rRNA processing [19]
NPM1	deacetylation	ageing [20]
PGK1	deacetylation	glycolysis [21]
GABP*β*1	deacetylation	mitochondrial homeostasis [17,22,23]
U3-55k	deacetylation	pre-rRNA processing [24]
H3K122	desuccinylation	chromatin compaction [15]
FOXO3	deacetylation	monocyte apoptosis [25]
FKBP51	deacetylation	Akt activity [26]
CDK9	deacetylation	RNA polymerase II transcription [27]
DDB1	deacetylation	activity of the CUL4B/DDB1/DCAF1 E3 ubiquitin ligase complex [28]
DDX21	deacetylation	transcription elongation and genome stability [29]
SMAD4	deacetylation	breast cancer metastasis [30]
OSX	deacetylation	bone formation [31]
WDR77	deacetylation	transmethylase activity of the WDR77/PRMT5 complex [32]
Fibrillarin	deacetylation	rRNA synthesis [33]
H3K36/K37	deacetylation	heterochromatin silencing [34]
ATM	deacetylation	DNA repair [35]
Ran	deacetylation	nuclear export of NF-κB p65 [36]
H4K91	deglutarylation	chromatin structure [16]
GATA4	deacetylation	stress-induced cardiac hypertrophy
STRAP	deacetylation	p53 activity and stability [37]
CRY1	deacetylation	circadian phase coherence and glucose homeostasis [38]
Nfatc1	deacetylation	hair growth [39]
USP39	deacetylation	hepatocellular carcinoma development [40]

In chromatin, SIRT7 selectively deacetylates histone H3 lysine 18 (H3K18Ac), which serves to maintain the cellular transformation ability of human cancer cells and tumour formation *in vivo* [14]. SIRT7 also functions as a desuccinylase of histone H3 lysine 122 and a deglutarylase of histone H4 lysine 91 to promote chromatin compaction [15,16]. Despite its prominent roles regulating chromatin, SIRT7 also deacetylates several non-histone proteins, including U3-specific protein U3-55 k and nucleolar organizer polymerase-associated factor 53 (PAF53) that is involved in the precursor ribose RNA (pre-rRNA) processing [19,24]. SIRT7 also deacetylates GA-binding protein β1 (GABPβ1) to regulate mitochondrion function and phosphoglycerate kinase 1 (PGK1) in regulating glycolysis [21,22]. In addition, SIRT7 participates in ageing processes and breast cancer lung metastasis by deacetylating nucleophosmin (NPM1) and SMAD4 [20,30]. SIRT7 also serves as a key activator of the telogen-to-anagen transition in cycling hair follicles; here, it acts as the deacetylase of NFATc1, which helps activate dynamic hair follicle stem cells [39]. To further widen the range of SIRT7 deacetylation targets, our laboratory conducted stable isotope labelling in SIRT7 knockout cell line coupled with quantitative mass spectrometry. We found a comprehensive list of candidates involved in a variety of functions, ranging from gene regulation to chromatin architecture homeostasis and metabolism [41].

Moreover, multiple studies reveal that SIRT7 regulates proteostasis/endoplasmatic reticulum (ER) stress, mitochondrial protein folding stress and mitochondrial metabolism [17,22,23,42]. SIRT7 is recruited to the promoters of ribosomal protein genes via transcription factor *Myc* to repress gene expression and to alleviate ER stress [42]. In addition, SIRT7 inactivation caused reduced quiescence, increased mitochondrial protein folding stress, and expression of SIRT7 is reduced in aged haematopoietic stem cells (HSCs) [23]. The same phenomenon was observed in human haematopoietic cells [43]; conversely, SIRT7 upregulation significantly improved the regenerative capacity of aged HSCs [23]. This is the first report linking stem cell ageing and SIRT7, giving the hope for targeting the dysregulated cellular programme to reverse HSC ageing. SIRT7 deacetylates GABPβ1, an important role of regulator of nuclear-encoded mitochondrial genes, which impacts mitochondrial function [22]. SIRT7 arginine methylation, which inhibits its H3K18 deacetylase activity, mediated glucose sensing and signalling with mitochondria biogenesis to maintain energy balance [17].

Most notably, SIRT7 is a crucial player in the DDR: it has histone deacetylase activity at DNA damage sites and exhibits other catalytic activities towards proteins involved in DNA damage and repair [15,35,44]. We discuss these processes in more detail below.

## 3. SIRT7 in maintaining genome stability

### 3.1. SIRT7: guardians of genome integrity and stability

**Figure 2.**
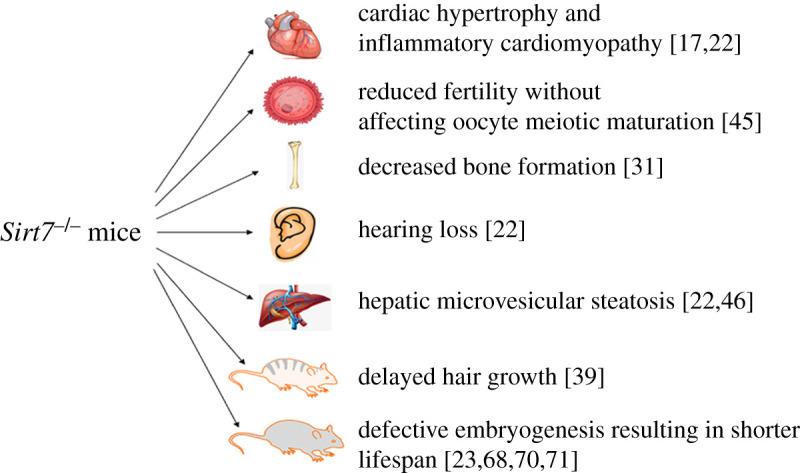
SIRT7 knockout mice phenotypes.

Numerous studies support a role for SIRT7 in genome stability and organismal viability. Much support has come from the use of *Sirt7* knockout mice (figure 2). Vakhrusheva *et al.* [17] found that *Sirt7*-deficient mice suffer from degenerative heart hypertrophy, accompanied by inflammatory cardiomyopathy and decreased resistance to cytotoxic and oxidative stress. In female *Sirt7* knockout mice, Vazquez *et al.* [45] found that *Sirt7^−/−^* females exhibit reduced fertility without an effect on oocyte meiotic maturation. Multisystemic mitochondrial dysfunction is also observed in *Sirt7-*deficient mice.

SIRT7 functions at chromatin to suppress ER stress and prevents fatty liver disease, and SIRT7-deficient mice develop chronic hepatosteatosis resembling human fatty liver disease, and liver-specific reconstitution of SIRT7-deficient mice reversed the fatty liver phenotype. Strikingly, SIRT7 overexpression in the livers of high-fat, diet-fed mice suppressed ER stress and rescued the fatty liver phenotype [42]. *Sirt7^−/−^* pups are born at sub-Mendelian ratios, indicating a defect in embryogenesis. Mutant mice that survive to adulthood exhibit a shortened lifespan with signs of accelerated ageing such as premature (6 months), kyphosis and decreased gonadal fat pad content [44]. *Sirt7^−/−^* mice exhibit elevated blood lactate levels, exercise intolerance, cardiac dysfunction, microvesicular steatosis and age-related hearing loss. In addition, in the liver-specific *Sirt7* KO (*Sirt7^hep−/−^*) mice display the same hepatic mitochondrial dysfunction and represents SIRT7 activity in a cell-autonomous effect on mitochondria function [22]. SIRT7 expression is reduced in aged HSCs, which are characterized by increased apoptosis, loss of quiescence and decreased reconstitution capacity, features resembling those observed in *Sirt7^−/−^* mice, and in mice reconstituted with *Sirt7^−/−^* HSCs improved their regenerative capacity [23].

Using hair follicle stem cell-specific *Sirt7* knockout mice, Li *et al.* [39] found that loss of *Sirt7* impedes the follicle life cycle transition from telogen to anagen phase and delays hair growth. In addition, in response to pressure overload, the cardiomyocyte-specific *Sirt7* knockout mice show severe cardiomyocyte hypertrophy [46]. Osteopenia-specific *Sirt7* knockout mice showed decreased bone formation that occurred via acylation of SP7/Osterix (OSX)—a transcription factor that activates genes involved in osteoblast differentiation [31]. Finally, Fang *et al.* [47] reported that *Sirt7*-deficient mice show increased *Sirt1* activity, resulting in inhibited PPARγ expression and thus restrained adipocyte differentiation and diminished white fat accumulation. The phenotypic consequences of SIRT7 deficiency could be explained by the functional link of SIRT7 with the maintenance of genome stability.

### 3.2. SIRT7 regulates DNA double-strand break repair

DNA double-strand breaks (DSBs) constitute the most toxic type of DNA lesion. As such, they must be efficiently repaired to maintain genome stability. DSBs are mainly repaired by NHEJ, which is predominant in non-cycling cells exposed to genotoxic stress, and HR, which functions in proliferating cells as it requires the pairing of sister chromatids [48]. Regarding HR, data from a previous report suggested that SIRT7 might regulate HR-mediated repair [49]. However, the detailed mechanism remains largely unknown and requires further investigation. For this reason, we explain how SIRT7, which is efficiently recruited to DSBs, is involved in mediating NHEJ. Whether SIRT7 is involved in other forms of DNA repair is largely unknown.

Upon DSBs, driven by a signalling cascade, which is initiated by ataxia-telangiectasia mutated (ATM)-mediated phosphorylation of histone 2A variant H2AX to generate γ-H2AX, this process is followed by the recruitment of the mediator of DNA damage checkpoint protein 1 (MDC1) and activation of RNF8–RNF168-dependent ubiquitination. Following the ubiquitination of H2A at lysine 13 and lysine 15 (H2AK13ub and H2AK15ub), and histone H4 lysine 20 dimethylation (H4K20me2) and histone H4 lysine 16 monomethylation (H4K16me1), 53BP1 is rapidly recruited onto chromatin surrounding the DSBs where it serves as an effector of the NHEJ pathway [50–54].

**Figure 3.**
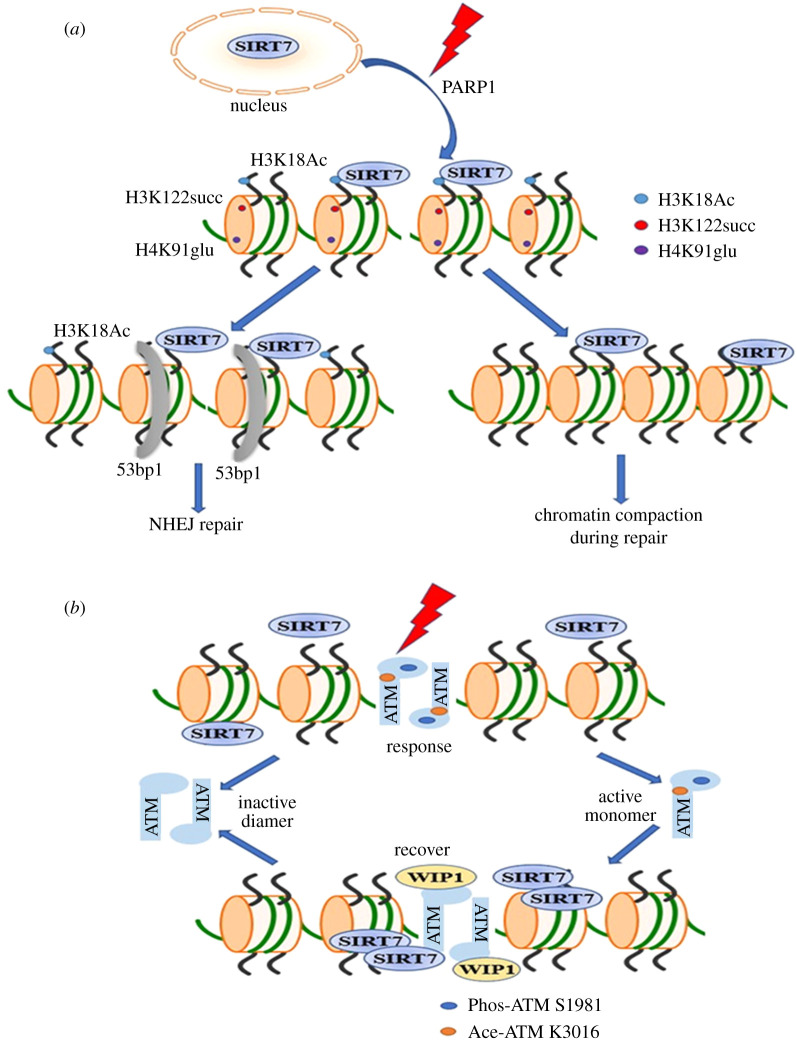
The role of SIRT7 in DNA repair. (*a*) In response to DNA damage, SIRT7 is recruited to DSBs that depend on PARP1, where it deacetylates H3K18ac and allows 53bp1 recruitment for the repair via the NHEJ pathway. After its recruitment to chromatin, SIRT7 mediates H3K122 desuccinylation and H4K91 deglutarylation for chromatin compaction necessary for DNA repair. (*b*) In response to DNA damage, ATM is sequentially modified by acetylation and phosphorylation before ATM dissociates into an active monomer. At the late stage of DNA damage and repair, SIRT7 is gradually recruited to DNA damage sites and deacetylates ATM; this process facilitates ATM dephosphorylation by WIP1. After complete repair, ATM dimerizes into an inactive form.

Interestingly, Vazquez *et al.* [44] found that 53BP1 foci are remarkably reduced in SIRT7^−/−^ cells, and that DNA damage, mutations and replication stress accumulate. The resulting genome instability leads to compromised NHEJ (figure 3*a*).

SIRT7 is, in fact, recruited to DSBs, but at a relatively slower rate compared with SIRT1 and SIRT6 [44]; its recruitment depends on poly (ADP-ribose) polymerase (PARP) activity, which ensures the recruitment of several DNA damage repair proteins to damaged sites [44,55,56]. A direct interaction between SIRT7 and PARP1 has been reported [15], but the detailed mechanism and function of SIRT7–PARP1 interplay is unknown.

Consistent with the previous report regarding the effects of SIRT7 on NHEJ, Chen and coworkers identified the Dicer protein in the regulation of SIRT7 localization upon DNA damage. They find that DNA damage agents can induce Dicer expression and results in increased trapping of SIRT7 in the cytoplasm and increases H3K18 acetylation at sites of damaged DNA and facilitates NHEJ repair pathway [57,58]. Growing evidence supports the importance of chromatin modification at or around DNA-damaged sites in DDR [59,60]. Again, data provided by Vazquez *et al.* [44] showed that SIRT7-mediated H3K18 deacetylation affects 53BP1 recruitment to DNA damage sites. H3K18Ac is directly involved in DNA repair, and H3K18Ac levels are fine-tuned by SIRT7 in response to DNA damage. Meanwhile, Li *et al.* [15] showed that SIRT7 is recruited to DSBs and catalyses the desuccinylation of histone H3 lysine 122, thereby promoting chromatin condensation and efficient DSB repair. Bao *et al.* [16] demonstrated that endogenous *Sirt7* functions as a histone deglutarylase to regulate histone H4 lysine 91 glutarylation dynamics. In response to DNA damage, *Sirt7* depletion hindered the removal of H4K91glu. Similar to SIRT7-mediated H3K122 desuccinylation, the removal of H4K91 glutarylation also aims at promoting chromatin condensation for the DNA repair process [16] (figure 3*a*). It is of great interest that all three sites—H3K18Ac, H3K122succ and H4K91glu—are mediated by SIRT7 during DNA damage repair. Whether these sites function independently or in a synergistic manner is largely unknown. Further studies are warranted to shed light on how SIRT7-mediated epigenetic regulation collaborates with the functions of other repair proteins recruited to DSBs and the underlying regulatory network. However, based on the above findings, it is clear that SIRT7 is required during the early phase of DNA repair and that a signalling mechanism is deployed that links histone modification to DSB repair.

These findings establish the role of SIRT7 in the early phase of DNA repair and elucidate novel signalling that links histone modification and DSB-related repair. During the process of DNA damage and repair, the proteins recruited to DNA damage sites are gradually displaced and inactivated, which make the cells return to the normal state and ensure faithful DNA repair. Among the numerous key DNA damage response factors, ATM has been reported to be an apical kinase in response to DSBs. Through exposure to DNA damage, ATM is activated through a series of highly organized machineries [61–65]. Acetylation and phosphorylation are two key post-translational modifications involved in activation of ATM in response to DSBs, both are dynamically regulated. Our research fills the gap of the dynamic regulation of ATM acetylation, and we find that SIRT7 is gradually recruited to chromatin in the late phase of repair and deacetylate ATM, which is required for the dephosphorylation of ATM by the phosphatase WIP1, and thus ensure the faithful DNA repair [35] (figure 3*b*). How SIRT7 regulates the downstream of ATM signalling needs more exploration.

Interestingly, re-localization of SIRT7 from the nucleolus to DNA damage sites affects ribosomal transcriptional repression [44,66,67]. This finding suggests that SIRT7-mediated DNA repair might have consequences on genome-wide transcriptional regulation under conditions of chronic DNA damage, plausibly the restoration of transcriptional profiles.

On the other hand, R-loop is a three-stranded nucleic acid structure; its aberrant formation and persistence cause DNA damage. Song *et al.* [29] showed that SIRT7-mediated deacetylation of DDX21 deacetylation cooperates which helps to prevent R-loop accumulation and DSBs, thus safeguarding genome integrity.

### 3.3. Role of SIRT7 in cancer and ageing

Increased genome instability is a common hallmark of both ageing and cancer. Consequently, any defect in DNA repair can contribute to genomic instability and subsequently lead to accelerated ageing or tumorigenesis [68,69]. DNA damage accumulates with age, and defects in DNA repair can cause phenotypes of premature ageing. Below, we describe the emerging data that suggest defects in SIRT7-mediated genome stability can affect ageing.

A longevity function has been proved for mammalian sirtuins. Indeed, *Sirt7*-deficient mice exhibit a reduction in mean and maximum lifespans, which indicates the role of SIRT7 in the ageing process [17]. By performing a comparative interactomics study associated with DNA repair, chromatin assembly and ageing, Lee *et al.* [20] found that SIRT6 and SIRT7 regulate NPM1 during the ageing process.

As mentioned earlier, researchers offered insights into the role of SIRT7 in the ageing process, showing that SIRT7 protects adult hair follicle stem cells from ageing by ensuring their progression through the hair growth cycle [39,70]. Bi *et al.* [71] also delineated the mechanisms of human stem cell ageing, showing that SIRT7 can form a complex with the nuclear lamina and heterochromatin proteins to maintain a repressive heterochromatin state and regulate the innate immune response during stem cell ageing. Moreover, Liu *et al. [72]* showed that SIRT7 deficiency leads to lowered histone acetyltransferase 1 (HAT1) activity and decreased H4K5 and H4K12 acetylation, which affects chromatin assembly. They also obtained evidence that SIRT7 ablation results in aneuploidy and ageing phenotypes, including senescence and nucleolar expansion [72].

Genomic instability in rDNA repeat sequences is an underlying cause of cell ageing [68]. Paredes *et al.* [73] uncovered an important role for SIRT7 in guarding against rDNA instability and protecting against senescence through association with SNF2H. Taken together, it seems that SIRT7 serves as an important regulator of mammalian longevity and might act as a molecular bridge between ageing and genome stability, paving the road for use. These preliminary findings offer support to investigate the value of targeting SIRT7 in the treatment of age-related diseases.

Based on the studies of SIRT7 in cancer, Kiran *et al.* [74] demonstrate that SIRT7 plays an important role in cell survival of osteosarcoma (U2OS) under DNA damage-induced stress. Specifically, the researchers showed that SIRT7 attenuated the effects of genomic stress, as SIRT7 knockdown cells showed increased susceptibility to the DNA damaging agent doxorubicin. Mechanistically, the cell cycle of SIRT7-overexpressing cells is temporarily halted at the G1/S phase when DNA damage is detected, probably to ensure DNA repair. SIRT7 resulted in reduced accumulation of γ-H2AX, p53 and the attenuation of stress-activated protein kinases (p38 and JNK) to maintain the genome integrity [74]. Beside the role of SIRT7-mediated H3K18 deacetylation in maintaining a malignant phenotype, Pandey & Kumar [75] provided evidence that HBx-dependent accumulation of SIRT7 favours H3K18 deacetylation and downregulation of RPS7, which is involved in the DDR and cancer cell transformation. Finally, data from our laboratory support that SIRT7 has degraded in response to 5-fluorouracil treatment and renders colorectal cancer cells sensitive to radiation [76]. The identification of SIRT7 inhibitors could thus be of great importance with respect to cancer treatment.

## 4. Conclusion

SIRT7 is involved in diverse cellular processes, including energy homeostasis, chromatin regulation, gene regulation and ribosome biogenesis. Here, we have highlighted the roles of SIRT7 in maintaining genome stability through its involvement in the DDR and the repair of DSBs. While it is clear that SIRT7 serves to promote DNA repair and ensure genome stability, how SIRT7 might interact with HR, MMR, NER and BER are still unclear.

While we know that SIRT7 regulates chromatin condensation in response to DNA damage via the desuccinylation of H3K122 and deglutarylation of H4K91 [15,16]. As a master epigenetic regulator, there are no doubt more epigenetic marks regulated by SIRT7 need to be studied for a comprehensive understanding of epigenetic regulation.

The importance of SIRT7 in DNA damage repair suggests that this enzyme might function as a tumour suppressor. However, SIRT7 is overexpressed in various cancers. Thus, SIRT7 might have opposing effects on cancer initiation and progression [14,32,76–78]. More systematic research is necessary to delineate how SIRT7 function might change across cancer evolution and development. A deeper understanding of SIRT7 function in genome stability at the molecular and physiologic levels may enable us to develop novel cancer- or ageing-related therapeutic targets. Such targets will be essential for conceptualizing the translation of SIRT7 biology into clinical applications.

## Data accessibility

This article does not contain any additional data.
